# Characteristics of smokers who have never tried to quit: evidence from the British Opinions and Lifestyle Survey

**DOI:** 10.1186/1471-2458-14-346

**Published:** 2014-04-11

**Authors:** Aarohi Sharma, Lisa Szatkowski

**Affiliations:** 1 Division of Epidemiology and Public Health, University of Nottingham, Clinical Sciences Building, Nottingham City Hospital, NG5 1 PB Nottingham, UK; 2 Division of Epidemiology and Public Health, UK Centre for Tobacco and Alcohol Studies, University of Nottingham, Clinical Sciences Building, Nottingham City Hospital, NG5 1 PB Nottingham, UK

## Abstract

**Background:**

An understanding of the characteristics of smokers who have never tried to quit may be useful to help identify and target these individuals and encourage them to attempt to give up smoking. Using national survey data we investigated variables associated with smokers reporting never having tried to quit.

**Methods:**

Using data from the 2007 and 2009 UK Office for National Statistics Opinions and Lifestyle Survey we identified all self-reported current smokers aged 16+. The primary outcome was response to the question ‘have you ever tried to quit smoking?’ Univariable and multivariable logistic regression quantified the association between this outcome and several potential explanatory variables, including age, sex, socioeconomic status, health status, smoking behaviour, and knowledge of the dangers of smoking.

**Results:**

Desire to quit was the most significant independent predictor of whether a smoker reported never having tried to quit. Smokers who reported that their health was good or very good were more likely to report never having tried to quit than those whose health was fair, bad or very bad (OR 1.59, 95% CI 1.05-2.41). Smokers who reported that no family members, friends or colleagues had been trying to get them to quit smoking in the last year were more likely to report never having tried to quit than those who reported that someone was trying to persuade them (OR 1.57, 95% CI 1.09-2.28). Smokers who hadn’t received any cessation advice from a health professional in the last five years which they considered to be helpful were also more likely to report never having tried to quit.

**Conclusions:**

Smokers who do not want to quit, who are in good health, whose friends and family are not trying to get them to quit, and who do not report receiving helpful advice to quit from a health professional, are more likely to report never having tried to quit.

## Background

Smoking remains a major burden on the health of individuals and populations, responsible for approximately 79,100 deaths in England in 2011 [1]. Though smoking prevalence has declined in Britain since the 1970s, there is evidence of a slowing in the rate of progress more recently [2]. The frequently-cited, though contentious, ‘hardening hypothesis’ has been proposed to explain this, suggesting that smokers who find it easiest to quit have already done so, leaving behind a group of continuing smokers who are resistant to quitting [3,4].

Previous work has sought to understand the factors associated with making a quit attempt. A study carried out in the USA reported that smokers were more likely to attempt to quit if they smoked fewer cigarettes per day, started smoking at a later age, had already made an attempt at quitting, were less dependent on nicotine, were facing greater pressure to stop smoking and had an expectation that they would quit in the near future [5]. However, this study used data from employees at a cancer treatment and research centre whose behaviour, if influenced by their place of work, may not be representative of all smokers. In addition, since the publication of this study in 1991 public attitudes towards smoking have changed considerably and a raft of tobacco control measures has been introduced in an attempt to reduce smoking prevalence. The variables reported in this study as risk factors for not attempting to quit may no longer hold true in this changed environment. More recent data have highlighted intention to quit, longer duration of previous quit attempts, younger age, negative attitudes about smoking, and lower levels of nicotine dependence as significant predictors of reporting having tried to give up smoking within the past year [6]. Agreement with the statements ‘I like being a smoker’ and ‘I enjoy smoking’ were reported as independent predictors of failing to make a quit attempt in a six month prospective study [7]. In a second prospective study of smokers who were not interested in quitting at baseline, self-reported motivation and self-efficacy to quit significantly predicted making a quit attempt during the six month follow-up period [8]. Experiencing financial stress has also been reported to be significantly associated with not trying to quit smoking over a one year follow-up period [9]. A systematic review synthesising existing literature found that having made a previous quit attempt and being motivated and intending to stop were the most dominant factors associated with making a quit attempt. Having a negative opinion about smoking, being worried about the effects of smoking on health and quality of life, expecting health benefits from quitting, having a ban on smoking in the home, and being confident of success in quitting also showed significant associations with making a quit attempt in a number of the studies identified for inclusion in the review [10].

To our knowledge few data exist which quantify the proportion of smokers who have never tried to quit and describe their characteristics. An understanding of these characteristics may be useful to identify and target smokers who have never tried to quit and encourage them to try to give up smoking. Given that cessation is a dynamic process and that many smokers will make many failed quit attempts before finally succeeding [11], it is crucial to encourage an attempt at cessation in smokers who might otherwise not try, in order to increase the likelihood of ultimate success.

This study uses recent data from the British Opinions and Lifestyle Survey, a repeated cross-sectional national survey, to examine the association between multiple potential risk factors and whether a smoker reports never having tried to quit smoking.

## Methods

### Data source

The UK Office for National Statistics (ONS) Opinions and Lifestyle Survey (formerly known as the ONS Opinions Survey and the ONS Omnibus Survey) is a multi-purpose, face-to-face, modular monthly survey of adults aged 16+ living in private households in Great Britain. Further details about the survey methodology are available elsewhere [12,13]. In two months in most years the survey has gathered information about respondents’ smoking behaviour; this study combines data from the October and November 2007 and February and March 2009 surveys, the two most recent surveys for which data were available at the time of writing. Current smokers were identified as those who responded positively to the question ‘do you smoke cigarettes at all nowadays’, and smokers who reported never having tried to quit were defined as those who responded negatively to the question ‘have you ever tried to quit smoking?’.

Guided by the questions asked in the Opinions and Lifestyle Survey, as well as existing literature, data on several variables potentially associated with reporting never having tried to quit were extracted, as shown in Table 1. These data included socio-demographic information (age, sex, NS-SEC social group [14], highest qualification attained, access to a car and household type); health data (self-rated health and self-reported presence of a longstanding illness, disability or infirmity); smoking behaviour (daily cigarette consumption, time between waking and smoking the first cigarette of the day, smoking rules in the home); and quitting behaviour (desire and intention to quit, persuasion by others to quit, receipt of advice to quit from a health care professional). Respondents’ knowledge of the dangers of smoking was assessed by whether they correctly identified smoking as the cause of most deaths before the age of 65 in the UK, from a range of possible answers including road accidents, accidents at work, AIDS, murder and manslaughter, illicit drugs and alcohol misuse. Knowledge of the dangers of passive smoking was quantified using responses to 12 questions which asked respondents whether living with a smoker and breathing someone else’s smoke increases children’s and non-smokers’ risks of developing a number of conditions (such as asthma, ear infections, diabetes, cot death, chest infection lung cancer and heart disease). The number of correct responses each participant gave was calculated, and the variable cut at the median total score to give a binary variable indicating knowledge above and below the sample median.

**Table 1:** Univariable odds ratios for the association between risk factors and reporting never having tried to quit smoking

**Variable**	**N**	**% never tried to quit**	**Odds ratio (95% CI)**	**Wald p-value**
**All respondents**	877	21.3		
**Sex**				
Female	448	21.4	1.00	0.918
Male	429	21.1	0.98 (0.68-1.41)	
**Age group**				
65+	133	18.0	1.00	0.017
55-64	133	20.2	1.15 (0.60-2.23)	
45-54	158	13.9	0.74 (0.37-1.47)	
35-44	181	20.3	1.16 (0.64-2.10)	
25-34	168	21.4	1.24 (0.67-2.29)	
16-24	104	35.1	2.47 (1.30-4.67)	
**NS-SEC social group**				
Managerial and professional occupations	226	16.8	1.00	0.080
Intermediate occupations	157	20.4	1.27 (0.72-2.26)	
Routine and manual occupations	431	21.8	1.39 (0.89-2.17)	
Not classified	63	33.7	2.52 (1.24-5.10)	
**Highest educational qualification**				
No formal qualifications	290	23.4	1.00	0.087
Level 1 GCSE D-G	54	20.6	0.85 (0.41-1.78)	
Level 2 GCSE A*-C	186	16.5	0.65 (0.38-1.10)	
Level 3 A levels or equivalent	110	31.5	1.50 (0.87-2.59)	
Level 4 At least some higher education	148	18.3	0.73 (0.41-1.32)	
Other qualifications	89	16.3	0.64 (0.33-1.23)	
**Does your household have access to a car?**				
Has access to a car	569	19.7	1.00	0.109
No access to a car	308	25.1	1.37 (0.93-2.00)	
**Household structure**				
One person only	294	22.9	1.00	0.079
Married cohabiting with dependent child	164	21.9	0.95 (0.58-1.54)	
Married cohabiting no dependent child	243	15.9	0.64 (0.40-1.00)	
Lone parent with dependent child	100	22.9	1.00 (0.53-1.88)	
Lone parent no dependent child	18	16.8	0.68 (0.18-2.49)	
Other	58	34.7	1.79 (0.94-3.40)	
**How good is your health?**				
Fair/bad/very bad	272	15.0	1.00	0.007
Good/very good	605	23.7	1.76 (1.16-2.67)	
**Do you have a longstanding illness?**				
Yes, and limits activities	220	17.3	1.00	0.040^*^
Yes, but does not limit activities	93	13.1	0.72 (0.33-1.58)	
Do not have illness/disability	564	24.0	1.51 (0.96-2.36)	
**Average daily cigarette consumption**				
10 or less	84	23.0	1.00	0.712
11 to 20	91	25.9	1.17 (0.53-2.58)	
21 to 30	214	21.5	0.92 (0.47-1.78)	
31+	488	19.9	0.83 (0.45-1.53)	
**How soon after waking do you smoke your first cigarette of the day?**				
After 60 minutes	318	24.1	1.00	0.249
30-59 minutes	171	17.3	0.66 (0.39-1.10)	
5-29 minutes	261	22.4	0.91 (0.59-1.40)	
Within 5 minutes	127	16.2	0.61 (0.32-1.14)	
**Which statement best describes the rules on smoking inside your home?**				
Smoking is not allowed at all	218	19.5	1.00	0.023
Smoking is allowed in some rooms or at some times	395	18.2	0.92 (0.58-1.46)	
Smoking is allowed anywhere	264	28.2	1.62 (1.00-2.62)	
**How much do you want to quit?**				
Very much indeed	218	6.69	1.00	<0.001
Quite a lot	209	15.5	2.56 (1.20-5.47)	
A Fair amount	129	20.2	3.52 (1.59-7.79)	
A little	67	21.0	3.71 (1.53-8.97)	
Don’t want to quit	254	38.7	8.82 (4.44-17.51)	
**When do you intend to give up smoking?**				
Within the next month	117	12.3	1.00	<0.001
Within the next 6 months	201	10.8	0.86 (0.39-1.92)	
Within the next year	157	11.2	0.90 (0.39-2.09)	
Some point after next year	156	23.3	2.16 (1.02-4.59)	
I have no intention of giving up	246	39.8	4.72 (2.38-9.35)	
**During the last year has a family member, friend or colleague been trying to get you to quit smoking?**				
Yes	469	15.2	1.00	<0.001
No	408	28.8	2.25 (1.54-3.28)	
**In the last 5 years have you been given advice on smoking by a health professional and did you find it helpful?**				
Yes, I had a helpful discussion	266	10.1	1.00	<0.001
Yes, I was given something helpful to take away and read	44	14.5	1.51 (0.50-4.51)	
Yes, I had a discussion but didn’t find it useful	143	25.3	3.02 (1.59-5.74)	
Yes, I was given something to take away and read but didn’t find it useful	65	23.3	2.70 (1.23-5.94)	
No, I didn’t receive any advice	359	28.6	3.57 (2.09-6.11)	
**Did the respondent identify smoking as the cause of most deaths before the age of 65 in the UK?**				
Yes, correct response given	330	17.3	1.00	0.046
No, incorrect response given	547	23.6	1.48 (1.01-2.17)	
**How good is your knowledge about the dangers of passive smoking?**				
Above average	506	18.9	1.00	0.079
Below average	371	24.5	1.39 (0.96-2.00)	

OR = odds ratio, 95% CI = 95% confidence interval.

^*^Adjusted Wald p-values accounting for survey design may appear significant event though the confidence intervals for each category do not.

All data were obtained from the UK Data Service [13], and no additional ethical approval was required for their use.

### Statistical analysis

All analyses were carried out using STATA 12.0 (STATA Corp, College Station, TX). The Opinions and Lifestyle Survey uses a complex sampling methodology in which households are randomly selected from across the UK and then one person is randomly selected from each household for interview. A weighting variable is provided with the dataset which corrects for the unequal probability of selection, and we used Stata’s survey commands to account for this survey design in all analyses. Initially, univariable logistic regression was used to investigate the unadjusted association between each explanatory variable and whether smokers reported never having tried to quit. All variables deemed significant risk factors in the univariable analysis were initially included in a multivariable model. We then used non-automated backwards stepwise procedures with a cut-off p value of 0.05 to build a parsimonious adjusted model, with adjusted Wald probability values (compatible with Stata’s survey commands) used to determine the significance of each variable in the final model. We examined the variance inflation factor to check for collinearity between variables included in the final multivariable model.

## Results

From the combined survey response of 4,413 interviews in 2007 and 2009, 948 respondents reported smoking (20.8% accounting for survey design, 95% CI 19.5-22.1). One respondent did not state whether they had ever tried to quit, giving useable data from 947 people. 70 respondents (7.4%) did not have data available for one or more variables and so these people were excluded from analysis, leaving a sample of 877 current smokers with answers available across all potential explanatory variables. As a sensitivity analysis we compared our results from this complete case analysis with those from models in which missing data for each categorical variable were coded as a separate category; the results did not differ and so for brevity we present only the results from the complete case analysis here.

Our final sample consisted of 877 current smokers, of whom 184 (21.3%, 95% CI 18.4-21.5) reported that they had never tried to give up smoking. Table 1 shows the univariable association between each of the potential explanatory variables and the outcome of reporting never having tried to quit smoking.

As Table 1 indicates, several variables showed a statistically significant univariable association with reporting never having tried to quit smoking. Younger smokers were more likely to report they have never tried to quit, as were smokers in good or very good health. Respondents living in houses where smoking was allowed anywhere were more likely to report not having tried to quit compared to those living in houses where smoking was not allowed at all. Reporting not having tried to quit was also more likely amongst smokers who do not want to quit, had no intention of giving up or of giving up in the next year, and where nobody had been trying to get them to give up. Smokers who haven’t received any cessation advice from a health professional in the last five years, and those who did not find any advice or leaflets they were given to be useful, were approximately three times more likely to report not having tried to quit than those who reported receiving a helpful intervention.

Table 2 shows the full multivariable model indicating the variables which were independent risk factors for reporting never having tried to quit smoking. There was strong collinearity between respondents’ self-rated health and reported presence of a longstanding illness, but only the former was significant in the final multivariable model. Examination of the variance inflation factor suggested no collinearity between any variables included in the final model.

**Table 2:** Multivariable odds ratios for the association between risk factors and reporting never having tried to quit smoking

**Variable**	** Adjusted OR (95% CI)**	**Wald p-value**
**How good is your health?**		
Fair/bad/very bad	1.00	0.027
Good/very good	1.59 (1.05-2.41)	
**How much do you want to quit?**		
Very much indeed	1.00	<0.001
Quite a lot	2.25 (1.15-4.40)	
A Fair amount	3.16 (1.56-6.40)	
A little	3.31 (1.49-7.36)	
Don’t want to quit	6.97 (3.77-12.87)	
**During the last year has a family member, friend or colleague been trying to get you to quit smoking?**		
Yes	1.00	0.017
No	1.57 (1.09-2.28)	
**In the last 5 years have you been given advice on smoking by a health professional and did you find it helpful?**		
Yes, I had a helpful discussion	1.00	0.003
Yes, I was given something helpful to take away and read	1.26 (0.46-3.41)	
Yes, I had a discussion but didn’t find it useful	2.59 (1.42-4.71)	
Yes, I was given something to take away and read but didn’t find it useful	2.10 (0.98-4.51)	
No, I didn’t receive any advice	2.69 (1.60-4.52)	

OR = odds ratio, 95% CI = 95% confidence interval; ORs are mutually adjusted for the other variables included in the table.

In the multivariable model, desire to quit was the most significant independent predictor of whether a smoker reports never having tried to quit. Smokers who reported that their health was good or very good were more likely to report never having tried to quit than those whose health was fair, bad or very bad (OR 1.59, 95% CI 1.05-2.41). Smokers who reported that no family members, friends or colleagues had been trying to get them to quit smoking in the last year were also more likely to report not having tried to quit than those who reported that one of these had been trying to persuade them (OR 1.57, 95% CI 1.09-2.28). Smokers who haven’t received any cessation advice from a health professional in the last five years, and those who did not find any advice or leaflets they were given to be useful, were over twice as likely to report not having tried to quit than those who reported receiving a helpful intervention (thought the latter association was marginally non-statistically significant).

## Discussion

This study has highlighted four independent risk factors for smokers reporting never having tried to quit – self-reported health, desire to quit, the influence of other people in trying to prompt a cessation attempt, and whether cessation support perceived to be useful was received from a health professional.

Motivation to quit smoking is already known to be a significant risk factor influencing the chances of making a cessation attempt [11,15] and therefore it is unsurprising that a lack of desire to quit results here in an increase in the odds ratio for reporting never having made a cessation attempt. Similarly, it is unsurprising that smokers who reported their health to be good or very good were more likely to report never having tried to quit. The presence of health problems, particularly those directly attributable to smoking, is known to be a feature which prompts many smokers to evaluate their smoking behaviour and consider trying to quit [16].

This study highlights the important role that other people can play in ensuring a smoker tries to quit, be they healthcare professionals or friends and family members. Existing studies have shown that smokers who report being under pressure to stop are more likely to report making a quit attempt [5], and the results present here corroborate the opposite, that smokers who report that no one is encouraging them to stop are less likely to have tried to quit. Smokers cite receiving cessation advice from a physician as an important factor which can motivate them to quit [17]. Systematic review evidence suggests that six month quit rates among smokers who receive advice are one to three percentage points higher than unassisted cessation rates [18]. The results presented here suggest that whether a smoker perceives an intervention by a physician to be helpful is also an important factor associated with whether they report never having tried to quit. Smokers who hadn’t received any advice, or where their discussion with the healthcare professional or literature they were given were not perceived to be helpful, were over twice as likely to report never having tried to quit compared to those who reported a helpful discussion. There is some evidence that a helpful discussion with a healthcare professional is more strongly associated with making a quit attempt than the provision of literature to take away and read, even if the latter is also perceived as helpful. These results suggest that healthcare professionals should strive to deliver cessation interventions in a way that smokers will perceive as helpful, and should endeavour to check that the patient has understood the cessation message being conveyed to them.

As with all cross-sectional studies using survey data, this work has limitations. Data may be subject to recall and response biases and it is impossible to confirm the temporality of the association between risk factors and self-reported quit attempts. In addition, we acknowledge that many smokers may forget, and fail to report, quit attempts, particularly if the attempt was of short duration or was made a long time previously [19-21]. Despite these limitations, this study provides the most up-to-date information available about the characteristics of smokers who report never having tried to quit.

## Conclusion

Our results suggest a need to ensure cessation messages resonate with those smokers who are most likely to report never having tried to quit – the healthy, those who don’t want to give up smoking, and those who are not being encouraged to quit by other people. Healthcare professionals should be encouraged to deliver cessation support at every available opportunity. Further research is warranted to understand the appropriateness, feasibility and effectiveness of different ways of motivating the friends and families of smokers to encourage and support their loved ones to quit.

## Competing interests

The authors declared that they have no competing interests.

## Authors’ contributions

LS conceived the study. AS and LS performed the statistical analysis. Both authors wrote the paper and have read and approved the final manuscript.

## Pre-publication history

The pre-publication history for this paper can be accessed here:

http://www.biomedcentral.com/1471-2458/14/346/prepub
